# Inhibition of Snail Family Transcriptional Repressor 2 (SNAI2) Enhances Multidrug Resistance of Hepatocellular Carcinoma Cells

**DOI:** 10.1371/journal.pone.0164752

**Published:** 2016-10-19

**Authors:** Xin-Yu Zhao, Lei Li, Xiao-Bo Wang, Rong-Jie Fu, Ya-Ping Lv, Wei Jin, Chao Meng, Guo-Qiang Chen, Lei Huang, Ke-Wen Zhao

**Affiliations:** 1 Key Laboratory of Cell Differentiation and Apoptosis of Ministry of Education of China, Department of Pathophysiology, Shanghai Jiao Tong University School of Medicine (SJTU-SM), Shanghai, China; 2 Institute of Health Sciences, Shanghai Institutes for Biological Sciences of Chinese Academy of Sciences and SJTU-SM, Shanghai, China; 3 Ren-Ji Hospital, Shanghai Jiao Tong University School of Medicine, Shanghai, China; Icahn School of Medicine at Mount Sinai, UNITED STATES

## Abstract

China accounts for almost half of the total number of liver cancer cases and deaths worldwide, and hepatocellular carcinoma (HCC) is the most primary liver cancer. Snail family transcriptional repressor 2 (SNAI2) is known as an epithelial to mesenchymal transition-inducing transcription factor that drives neoplastic epithelial cells into mesenchymal phenotype. However, the roles of endogenous SNAI2 remain controversial in different types of malignant tumors. Herein, we surprisingly identify that anchorage-independent growth, including the formation of tumor sphere and soft agar colony, is significantly increased when SNAI2 expression is inhibited by shRNAs in HCC cells. Suppression of SNAI2 suffices to up-regulate several cancer stem genes. Although unrelated to the metastatic ability, SNAI2 inhibition does increase the efflux of Hoechst 33342 and enhance multidrug resistance *in vitro* and *in vivo*. In agreement with this data, we demonstrate for the first time that decreasing SNAI2 level can transcriptionally upregulate several ATP binding cassette (ABC) transporter genes such as ABCB1. Moreover, ABC transporters’ inhibitor verapamil can rescue the multidrug resistance induced by SNAI2 inhibition. Our results implicate that SNAI2 behaves as a tumor suppressor by inhibiting multidrug resistance via suppressing ABC transporter genes in HCC cells.

## Introduction

Snail family transcriptional repressor 2 (SNAI2), also known as SLUG, belongs to the highly conserved Snail/Scratch superfamily, which includes SNAI1 (SNAIL), SNAI3 (SMUC), and SCRTs etc.[1]. Mammalian SNAI2 has C_2_H_2_ type zinc fingers in its carboxyl-terminal region and highly conserved SNAG (Snail/Gfi) domain in the amino-terminal region [1–3]. SNAI2 binds to the E-box-containing promoter of its downstream target genes through its C-terminal, and acts as a transcriptional repressor depending on the N-terminal SNAG domain that interacts with co-repressors [4, 5]. E-cadherin is one of the well-known target genes negatively regulated by SNAI2. Since E-cadherin is indispensable in the maintenance of epithelial status, SNAI2 is regarded as inducers in epithelial to mesenchymal transition (EMT) in embryogenesis and tumorigenesis [3, 6–9]. SNAI2 is not required for mesoderm formation or for neural crest generation and migration as SNAI1 [10], but SNAI2 plays essential roles in melanocytes, hematopoietic stem cells, adipocytes and germ cells [11–14]. What’s more, SNAI2 is found to be critical in γ-irradiation-induced apoptosis, G1/S transition, and genome instability [15–17].

Based on different experimental system and cell context, the roles of SNAI2 protein in tumorigenesis and metastasis remain largely controversial. SNAI2 is regarded as a cancer promoter factor due to the following facts: SNAI2 promotes survival through suppression of apoptosis and drives EMT transition and metastasis, [7, 18–20]; SNAI2 blocks TKI-induced apoptosis in myeloid leukemia, lung cancer and neuroblastoma cells [21–23]; SNAI2 has been shown to drive lung metastasis of immortalized and transformed melanocytic Mel-STR cells [7]. Using three-dimensional organoid culture combined with in vivo mammary ductal tree regeneration assay [24], SNAI2 and Sox9 have been revealed as master regulators in maintaining the normal mammary stem cell status in murine mammary epithelial cells, and ectopic over-expression of SNAI2 together with Sox9 promotes the tumor-initiating ability of human breast cancer cells [18].

However, SNAI2 could behave as a tumor suppressor through inhibiting proliferation, driving cell differentiation, and repressing tumor-initiation [25, 26]. Liu J. et al [25] have reported that SNAI2 inhibits the proliferation of prostate cancer cells via reduction of Cyclin D1 expression. Caramel J. et al [26] have disclosed that SNAI2 is expressed in normal melanocytes and loss of SNAI2 staining is correlated with malignant level in human melanoma tissue samples. Recently, Ye X. et al [27] have found a marked reduction of endogenous SNAI2 expression in the initially formed hyperplastic lesions compared to normal glands in MMTV-PyMT transgenic model of mammary tumor. When tumor progressed to high-grade carcinomas, SNAI2-expressing cells remain to local instead of detaching and metastasis. SNAI2 expression is down-regulated in some of the pulmonary metastatic advanced carcinomas. Cells expressing high level of SNAI2 are deficient in tumor-initiating ability. So it remains largely unknown the roles of endogenous SNAI2 in different malignant tumor under diverse circumstances.

Liver cancer is the second leading cause of cancer death worldwide among men, and hepatocellular carcinoma (HCC) is the most primary liver cancers throughout the world. China accounts for about half of the total number of liver cancer cases and deaths [28, 29]. We analyzed the HCC cohort in Oncomine database and found SNAI2 expression is slightly up-regulated in 225 carcinoma tissues comparing to the 220 normal liver tissues (*P = 0*.*049*). To exploit the roles of endogenous SNAI2 in HCC cells, we demonstrate herein that knockdown of SNAI2 expression with shRNA lentivirus lead to enhanced anchorage-independent growth and up-regulated cancer stem cell gene expression in HCC cells. Moreover, SNAI2 knockdown decreases drug sensitivity of HCC cells to multiple chemotherapy drugs such as camptothecin, doxorubixin, epirubicin, and sorafenib *in vitro* and induces xenograft growth during camptothecin treatment *in vivo*, even though it has no effects on metastasis. Notably, silence of SNAI2 increases drug efflux pump activity and transcriptionally up-regulate ABC transporter genes such as ABCB1. Pretreatment with ABC transporter inhibitor can abolish the drug resistance induced by SNAI2 inhibition. These data indicate that SNAI2 suppress drug resistance through restraining the efflux pump activity of ABC transporters. Our study suggests that SNAI2 influences drug sensitivity in HCC cells, which will shed new insights on mechanisms of multidrug resistance of hepatocellular carcinoma.

## Materials and Methods

### Cell lines and reagents

Human immortal hepatic HL-7702 cells and HCC cell lines MHCCLM3 and SMMC-7721were obtained from the cell bank of Shanghai Institutes for Biological Sciences (Shanghai, China). Both cell lines were cultured in Dulbecco’s modified eagle medium (DMEM, GE Healthcare Life Sciences, Chicago, IL, USA) supplemented with 10% fetal bovine serum (FBS, Gibco, Grand Island, NY, USA), 100 U/ml penicillin and 100 μg/mL streptomycin, and grown in a 95% air and 5% CO_2_ humidified atmosphere at 37°C. Camptothecin (CPT) (TCI chemicals, Shanghai, China), sorafenib (Selleck, TX, USA), doxorubicin (DOX), epirubicin (Epi) and verapamil (Sigma-Aldrich, St. Louis, MO, USA) were dissolved in DMSO as stocking solution respectively, then diluted in sterile saline before use.

### shRNA design and viral infection

GV298-CMV-mU6-MSC-Cherry-Puromycin lentiviral plasmids against SNAI2 (shSNAI234 and shSNAI237) and control were purchased from GENECHEM (Shanghai, China). The target sequences for SNAI2 shown as following: 5’-CATTCTGATGTAAAGAAAT-3’ for shSNAI234, 5’-CGTATCTCTATGAGAGTTA-3’ for shSNAI237. These shRNA plasmids were co-transfected with packaging plasmids including pCMV-Δ8.91 and pMDG into HEK293T cells to produce cherry-tagged lentivirus. Forty-eight hours later, the viral supernatants were collected, filtered through 0.45 μm membrane (Merck-Millipore, Billerica, MA, USA) and added respectively into MHCCLM3 or SMMC-7721 cells incubated with the medium containing 1 μg/ml polybrene (sc-134220, Santa Cruz Biotechnology, Dallas, TX, USA). Stably expressed cells were selected by 1 μg/ml puromycin after viral infection for 48 hours.

### Plasmids and transfection

Lentiviral expression vector pLVX-IRES-tdTomato was purchased from Clontech Laboratories Inc. The pLVX-IRES-tdTomato-*SNAI2* plasmid was constructed by subcloning *SNAI2* cDNA from pCDNA3.1-SNAI2 plasmid (kindly provided by Prof. Qian Zhao, Shanghai Jiao Tong University School of Medicine) into pLVX-IRES-tdTomato vector, which was confirmed by DNA sequencing (Biosune, Shanghai, China). SNAI2 lentivirus was produced by co-transfecting pLVX-IRES-tdTomato-SNAI2 with packaging plasmids including psPAX2 and pMD2G into HEK293T cells. The viral supernatants were harvested and SMMC-7721 cells were infected. SMMC-7721 cells stably expressing SNAI2 were selected 48 hours after viral infection.

### Western blot

The whole cell lysates were extracted in 1×SDS buffer (2×SDS: RIPA = 1:1), equally loaded onto 12.5% or 7.5% SDS-PAGE, and subsequently transferred to the nitrocellulose membranes (Bio-Rad, Hercules, CA, USA). After blocking in 5% non-fat milk at room temperature for 1 hour, the membranes were incubated with the indicated primary antibodies overnight at 4°C, followed by HRP-linked secondary antibodies (Cell Signaling Technology, Beverly, MA, USA). The signals were detected by SuperSignal West Pico Chemiluminescent Substrate kit (Pierce, Rockford, IL, USA) according to the manufacturer’s instructions. Antibodies against SNAI2 (#9585), ABCB1 (#12683), E-cadherin (#3195) and cleaved caspase-3 (#9662) were purchased from Cell Signaling Technology. Anti-cleaved PARP-1 (sc-8007) antibody was purchased from Santa Cruz Biotechnology. Anti-β-actin antibody was purchased from Merck-Millipore.

### Tumor sphere culture

For tumor sphere formation [30], SMMC-7721 or MHCCLM3 cells were suspended into single cells and cultured in DMEM/F12 medium supplied with 1×B27 (Gibco-Life Technologies, Carlsbad, CA, USA), 0.4% BSA, 20 ng⁄mL EGF, 20 ng/mL bFGF, and 50 μg/mL insulin. Cells were seeded at 1000~2000 per well in 24-well ultra-low attachment plates (Corning, NY, USA). All tumor spheres were cultured at 37°C in a 5% CO_2_/95% air atmosphere. The number of spheres (>100 μm in diameter) was counted 10 days after seeding.

### Soft agar colony formation assay

Soft agar colony formation assay was performed as following: 2×10^4^ cells/mL cells in medium containing 0.3% low-melting-temperature agarose (Sangon Biotech, Shanghai, China), 3% FBS, 100 U/ml penicillin and 100 μg/mL streptomycin, were cultured on the top of the layer containing 0.8% agarose. The cells were incubated at 37°C for 7 days and colonies were monitored by microscope and calculated.

### RNA isolation and quantitative real-time RT-PCR (q-PCR)

Total RNA of cells was extracted by TriPure Isolation Reagent (Roche, Basel, Switzerland), followed by RNase-free DNase (Promega, Madison, WI, USA) treatment. Complementary DNA (cDNA) synthesis kit (Takara, Dalian, Liaoning, China) was utilized to synthesize cDNA according to the manufacturer’s instructions. Polymerase chain reaction (PCR) amplifications of the respective genes were carried out with Power SYBR Green PCR Master mix (Applied Biosystems, Warrington, UK) using the ABI PRISM 7300 system (Perkin-Elmer, Torrance, CA, USA). Each reaction was repeated at least three times independently. Sequences of PCR primers used in this study are shown in Table 1.

**Table 1:** Sequences of PCR primers.

Gene	Forward primer	Reverse primer
*ABCB1*	GGGATGGTCAGTGTTGATGGA	GCTATCGTGGTGGCAAACAATA
*ABCB11*	TTGGCTGATGTTTGTG GGAAG	CCAAAAATGAGTAGCACGCCT
*ABCC1*	CTCTATCTCTCCCGACATGACC	AGCAGACGATCCACAGCAAAA
*ABCC2*	CCCTGCTGTTCGATATACCAATC	TCGAGAGAATCCAGAATAGGGAC
*ABCC10*	GTCCAGATTACATCCTACCCTGC	GCCAACACCTCTAGCCCTATG
*ABCG2*	ACGAACGGATTAACAGGGTCA	CTCCAGACACACCACGGAT
*OCT4*	ACATCAAAGCTCTGCAGAAAGAACT	CTGAATACCTTCCCAAATAGAACCC
*NANOG*	GAATGAAATCTAAGAGGTGGCA	CCTGGTGGTAGGAAGAGTAAAGG
*EpCAM*	TGATCCTGACTGCGATGAGAG	CTTGTCTGTTCTTCTGACCCC
*ALDH1A1*	GCACGCCAGACTTACCTGTC	CCTCCTCAGTTGCAGGATTAAAG
*CD24*	CTCCTACCCACGCAGATTTATTC	AGAGTGAGACCACGAAGAGAC
*CD44*	CTGCCGCTTTGCAGGTGTA	CATTGTGGGCAAGGTGCTATT
*CD56*	GGCATTTACAAGTGTGTGGTTAC	TTGGCGCATTCTTGAACATGA
*CD133*	TTCTTGACCGACTGAGACCCA	TCATGTTCTCCAACGCCTCTT
*SNAI2*	CGAACTGGACACACATACAGTG	CTGAGGATCTCTGGTTGTGGT
*GAPDH*	TGGTATCGTGGAAGGACTCATGAC	ATGCCAGTGAGCTTCCCGTTCAGC

### Cell migration assays

#### Scratch wound healing assays

Cells as indicated were seeded in 6-well plates. After reaching 90% confluence, the cells were rinsed with DMEM without FBS and a wound was made by scratching the monolayer cells with a sterile plastic tip. Then, photos were taken immediately (as basic width) and 24 hours after wounded (as final width) by Olympus BX51 microscope equipped with a digital camera. The migration width of control cells was calculated as the width migrated (basic width minus final width) relative to the basic width. The relative cell migration ability of different groups was normalized to the control group.

#### Transwell cell migration assays

Transwell cell migration assays were performed as reported [31, 32]. Briefly, cells were trypsinized, resuspended with DMEM without FBS, and added to the upper chamber of 8-μm pore size transwell system (Becton Dickinson Labware, Bedford, MA, USA), while the lower chamber contained DMEM with 10% FBS. After incubation for 24 hours, cells that migrated to the lower surface of the filter membrane were fixed and stained with crystal violet. Migrated cells were photographed and counted under microscope.

#### xCELLigence RTCA assays

Cell migration assays were also performed using xCELLigence system with real-time technology as described [33]. Cell migration assays were also performed using xCELLigence system with real-time technology as described. Briefly, cells were added to the upper chamber in serum-free medium and the 10% FBS contained medium was added in the lower chamber of special CIM-plate 16 plates (Roche Diagnostics GmbH) using the RTCA DP instrument. The CIM-plates have the microelectrodes located on membranes of the upper chambers. Data was accessed and analyzed with RTCA software 1.2.

### Cell viability assay and IC50 values

To evaluate sensitivity of HCC cells to CPT, DOX, Epi and sorafenib, indicated cells were seeded in 96-well plates at a density of 8×10^3^/well (SMMC-7721) or 1.8×10^4^/well (MHCCLM3). After 24 hours, cells were incubated with vehicle control or different concentrations of CPT/DOX/Epi/sorafenib, in the presence or absence of 1 μmol/L verapamil for 48 hours. Then, cell counting kit-8 (CCK-8, Dojindo Molecular Technologies, Kumamoto, Japan) reagent was added into each well and incubated for 2 hours. Cell growth was measured by the absorbance at wavelength of 450 nm by Synergy H4 Hybred Reader (BioTek Instruments, Winooski, VT, USA) [32]. The half-maximal inhibitory concentration (IC50) values were calculated by nonlinear regression analysis using the GraphPad Prism 6.0 software (San Diego, CA, USA).

### FACS analysis

The cells were trypsinized and suspended at 1×10^6^ cells/mL in Hank’s balanced salt solution supplemented with 2% fetal bovine serum. These cells were then incubated with 5 μg/mL Hoechst 33342 (Molecular Probes, Invitrogen) at 37°C for 90 minutes. Cells were placed immediately on ice, washed and resuspended in cold HBSS containing 2% FBS. Propidium iodide (Molecular Probes, Invitrogen) at a final concentration of 1 μg/mL was added to the cells to gate viable cells. After filtered through a 40 μm cell strainer (BD Falcon), cells were sorted as Hoechst 33342 low or high staining populations using MoFlo carrying a double-laser (Beckman Coulter & Astrios EQ). Hoechst 33342 was excited with the UV laser at 350 nm and fluorescence emission was measured with 450/65 nm (Hoechst blue) and 670/30 nm (Hoechst red) optical filters.

### Luciferase assay

The sequence of upstream promoter [34, 35] of *ABCB1* was amplified by PCR from genomic DNA with primers F: 5’-AGCTCGAG CCCACAATACATACACAGATTCC-3’ and R: 5’-CCAAGCTTATCTGGTTGCTTCCTGAAGTG-3’. Then, luciferase reporter plasmid pGL3-*ABCB1*-luc was generated by cloning *ABCB1* promoter into pGL3-Basic vector (Promega). For luciferase assay, SMMC-7721 cells stably expressing shSNAI2/shControl were transfected with luciferse reporter plasmid pGL3-*ABCB1*-luc and pRLSV40-*Renilla*. Forty-eight hours after transfection, cells were collected and analyzed by the Dual-Luciferase Assay system according to the manufacturer’s instructions (Promega).

### Animal experiments

Six-week-old female BALB/c nude mice were purchased from Shanghai SLAC laboratory animal research center (Shanghai, China). Three million SMMC-7721 cells expressing shControl/shSNAI2 were subcutaneously inoculated in contralateral flanks of each mouse respectively. Two weeks after inoculation, the mice were randomly grouped and intraperitoneally injected with 20 mg/kg CPT. One CPT treatment cycle includes continuous three days’ injection and one day break, and the treatment period is four cycles. At the end of the treatment, mice were euthanized and tumors were dissected, photographed, and weighted. All animals were handled according to the ‘Guide for the Care and Use of Laboratory Animals’ and all animal experiments were approved by Experimental Animal Ethical Committee at the Shanghai Jiao Tong University School of Medicine.

### Statistical analysis

Analyses by the Student’s two-tailed *t*-test were performed using the GraphPad Prism 6.0 software. *P* values < 0.05 were considered significant.

## Results

### Inhibition of SNAI2 expression enhances anchorage-independent growth of HCC cells

**Fig 1:**
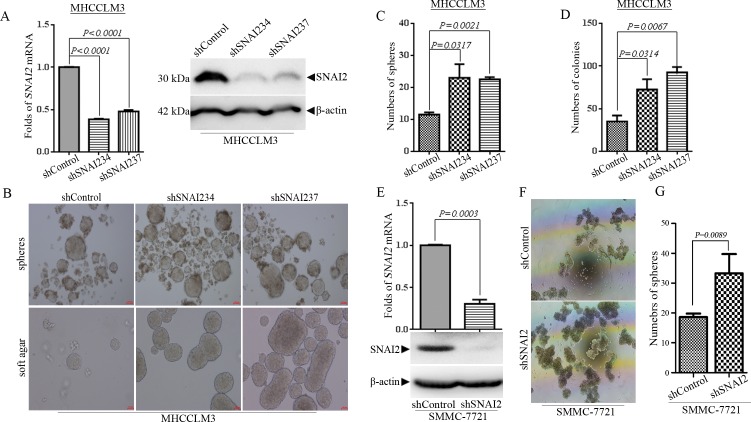
Inhibition of SNAI2 expression enhances anchorage-independent growth of hepatocellular carcinoma (HCC) cells. (A-D) MHCCLM3 cells were infected with lentivirus expressing shControl or shSNAI2s (shSNAI234/237) and selected by puromycin. (A) The expression of SNAI2 was detected by q-PCR (left) and Western blot (right). (B-D) Tumor sphere culture (B, up) and soft agar colony formation assay (B, down) were performed as described in Materials and Methods. Representative images of tumor spheres or soft agar colonies were shown (B), and the number of spheres (C) or colonies (D) (>100 μm in diameter) were counted and calculated by GraphPad Prism 6.0 software. (E-G) SMMC-7721 cells were infected with lentivirus expressing shControl or shSNAI2 and selected by puromycin. (E) The expression of SNAI2 was detected by q-PCR and Western blot. (F/G) SMMC-7721 cells were cultured under tumor sphere culture condition as in MHCCLM3 cells. Representative images of tumor spheres were shown (F) and the sphere number (>100 μm in diameter) were counted and analyzed by GraphPad Prism 6.0 software (G). (C, D, G) All values were represented as mean with bar as SD of three independent experiments and the *P* values were shown between two linked groups.

To investigate the roles of endogenous SNAI2 in HCC cells, we inhibited the expression of SNAI2 with two shRNAs, shSNAI234 and shSNAI237 in MHCCLM3 cells. The inhibition effect of shSNAI2s in mRNA and protein level were confirmed by q-PCR and Western blot respectively (Fig 1A), and the results illustrated that the expression of SNAI2 was attenuated effectively by introducing both shSNAI2s. Although SNAI2 inhibition could promote proliferation of non-cancer hepatic HL-7702 cells (S1A and S1B Fig), alteration of SNAI2 expression did not influence the proliferation of MHCCLM3 cells under regular culture conditions (data not shown), which was consistent with previous report [18]. Some have shown that mammary epithelial cells (MECs) with ectopic SNAI2 expression could endow anchorage-independent growth of MECs and generate more mammary organoids (spheres) than control group. To detect whether inhibition of endogenous SNAI2 could influence the anchorage-independent growth of HCC cells, MHCCLM3 cells were cultured under tumor sphere cultured (TSC) conditions [30] to compare their anchorage-independent growth. To our surprise, inhibition of SNAI2 increased instead of blocked the tumor sphere formation ability of HCC cells (Fig 1B-upper panel). MHCCLM3 cells expressing shSNAI2s had more tumor spheres than cells with shControl (Fig 1C) (*P<0*.*05*). Subsequently, colony formation assay in soft agar was measured as another form of anchorage-independent growth properties. Consistent with their growth under TSC conditions, shSNAI2-expressing MHCCLM3 cells possessed much higher colony formation activity, and the colony numbers doubled (*P<0*.*05*) in shSNAI234-expressing and tripled (*P<0*.*01*) in shSNAI237-expressing MHCCLM3 cells (Fig 1D). There were much larger colonies in shSNAI2 expressing groups than shControl counterparts (Fig 1B, lower panel). To exclude cell line specific effect, another HCC cell line SMMC-7721 cells were infected with shSNAI2 lentivirus, and SNAI2 expression was efficiently inhibited both in mRNA and protein levels (Fig 1E). Inhibition of SNAI2 expression significantly promoted the growth of tumor sphere of SMMC-7721 cells in TSC media as in MHCCLM3 cells (Fig 1F and 1G) (*P = 0*.*0089*). These results indicated that endogenous SNAI2 inhibited but not promoted anchorage-independent growth in HCC cells.

### Alteration of SNAI2 expression has no effect on metastatic properties of HCC cells

**Fig 2:**
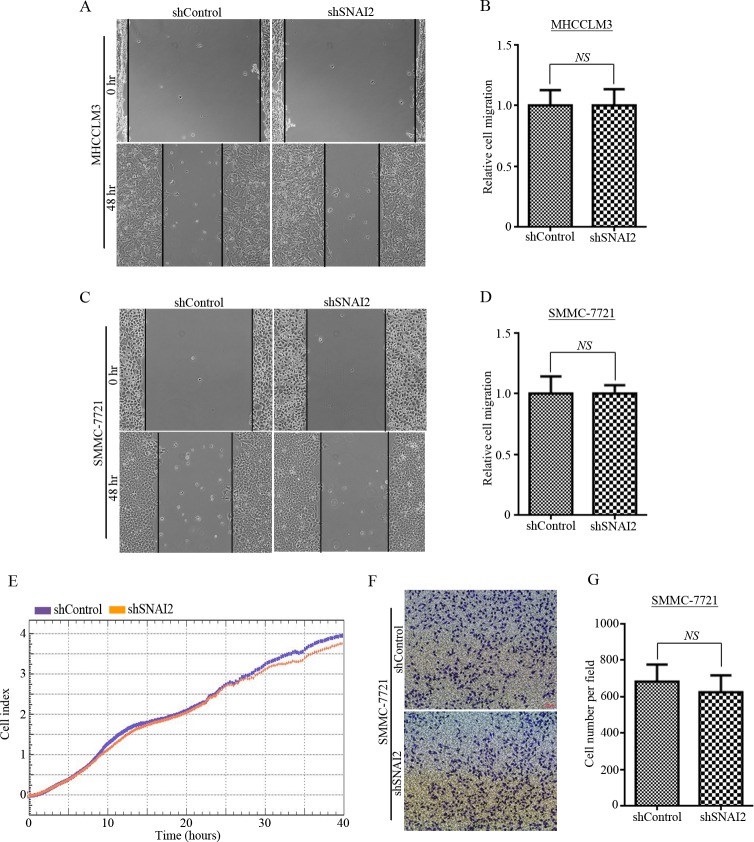
Alteration of SNAI2 expression has no effect on metastatic properties of HCC cells. (A-B) MHCCLM3 cells stably expressing shSNAI2 or shControl were used to test scratch wound healing activity (A), and relative migration distance was measured (B) and calculated by GraphPad Prism 6.0 software. (C-G) Metastatic abilities of SMMC-7721 cells expressing shSNAI2 or shControl were detected. Scratch wound healing ability was tested (C) and relative migration distance was measured and calculated by GraphPad Prism 6.0 software (D). Migration ability was investigated by xCELLigence RTCA assays (E) and in vitro transwell migration assay (F/G). Representative images of migration cells (F) were shown, and cell numbers were counted and analyzed by GraphPad Prism 6.0 software (G). All values were represented as mean with bar as SD of three independent experiments. All experiments were repeated at least three times with similar results.

SNAI2 was reported as a transcriptional factor that directly inhibits E-cadherin expression, which is a hall marker of EMT [3]. Consistent with previous report, over-expression of SNAI2 could effectively inhibit E-cadherin expression and SNAI2 inhibition could recover E-cadherin level in MHCCLM3 cells (S1C Fig). It has been reported that SNAI2 promotes the EMT process as well as metastatic phenotypes in several cancers as breast and lung cancer cells [19, 33, 36]. Then, we tried to figure out whether SNAI2 inhibition could influence metastatic features in HCC cells. Although the SNAI2 expression was blocked efficiently in MHCCLM3-shSNAI2 (shSNAI234) cells (Fig 1A, line 1/2), we did not detect any difference of migration ability between shControl and shSNAI2-expressing MHCCLM3 cells in scratch-wound healing assay (Fig 2A and 2B). Similar results were shown in SMMC-7721 cells: the scratch-wound healing ability did not change (Fig 2C and 2D), even though SNAI2 expression was significantly inhibited (Fig 1E). Moreover, xCELLigence RTCA assay (Fig 2E) and *in vitro* transwell migration assay (Fig 2F and 2G) were performed in shSNAI2-transfected SMMC-7721 cells with shControl-expressing cells as control. The results demonstrated that migration ability (Fig 2E, 2F and 2G) was not changed by SNAI2 suppression. Furthermore, we over-expressed SNAI2 in SMMC-7721 cells (S2A Fig). It is intriguingly to notice that over-expression of SNAI2 effectively inhibited the tumor sphere formation of SMMC-7721 cells (S2B and S2C Fig), which is consistent with previous results (Fig 1F and 1G). However, ectopic expression of SNAI2 did not influence migration ability of SMMC-7721 cells as accessed by scratch-wound healing assay (S2D and S2E Fig), xCELLigence RTCA assay (S2F Fig) and as well as *in vitro* transwell migration assay (S2G and S2H Fig). All these facts demonstrated that SNAI2 expression did not influence migration ability of HCC cells *in vitro*.

### SNAI2 inhibition is associated with upregulation of cancer stem cell (CSC) and ATP binding cassette (ABC) transporter genes in HCC cells

**Fig 3:**
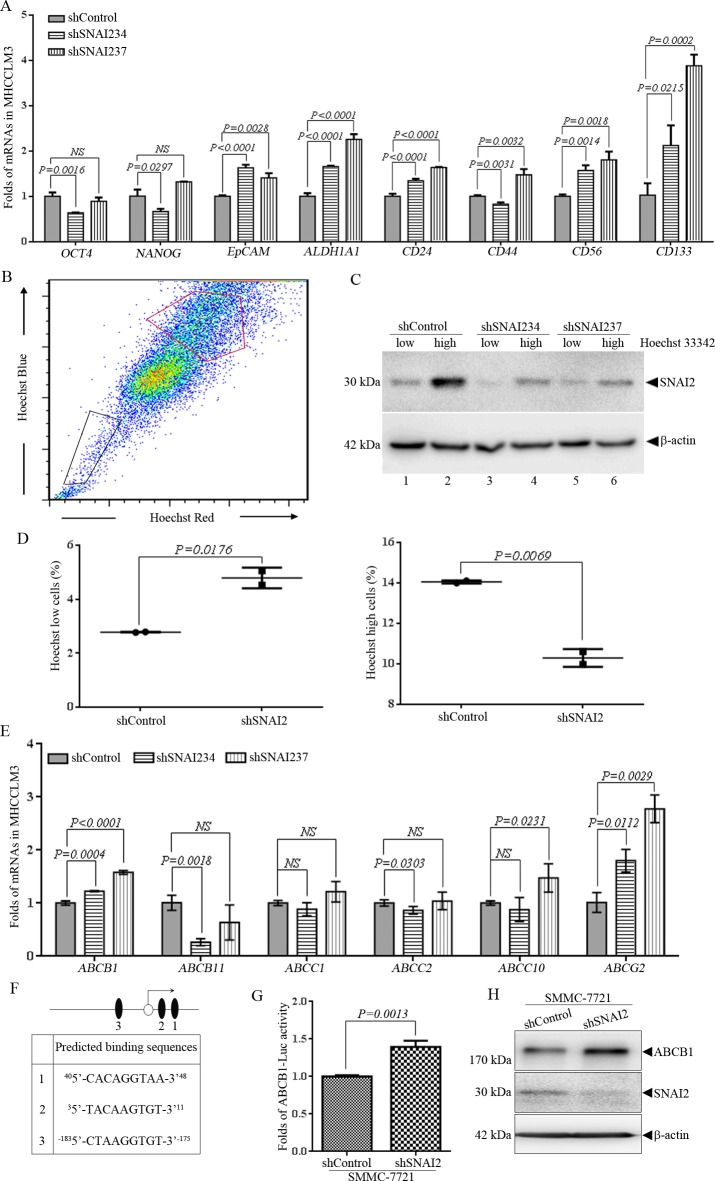
SNAI2 knockdown promotes the efflux of Hoechst 33342 and increases ATP binding cassette (ABC) transporter and CSC gene expression of MHCCLM3 cells. (A) Q-PCR was applied to detect mRNA levels of indicated genes in MHCCLM3 cells expressing shSNAI2/shControl shRNAs. Relative expression of indicated genes was normalized with *GAPDH* mRNA. (B-D) FACS was utilized to sorting cell populations of MHCCLM3 cells expressing shControl or shSNAI2 with low (black frame) and high (red frame) Hoechst 33342 staining (B), followed by Western blot for SNAI2 expression (C). Percentage of Hoechst low or high cell population was shown and compared (D). (E) Q-PCR was applied to detect mRNA levels of ABC transporter genes in MHCCLM3 cells expressing shSNAI2/shControl. Relative expression of ABC transporter genes was normalized with *GAPDH* mRNA. All values were represented as mean with bar as SD of three independent experiments, and the *P* values were measured between two linked groups. All experiments were repeated at least three times with similar results. (F) A diagram of the promoter of *ABCB1* gene that drives luciferase reporter plasmid pGL3-*ABCB1*-luc. Empty circle and black ovals represent the transcriptional start point of *ABCB1* gene and predicated binding sites of SNAI2 respectively. Sequences of predicated binding sites are shown in the text box below. (G) SMMC-7721 cells expressing shSNAI2 or shControl were transfected with luciferase reporter plasmid pGL3-*ABCB1*-luc and pRLSV40-*Renilla*. Luciferase Assay was conducted as described in Materials and Methods. (H) Western blot was applied to measure expression of indicated proteins in SMMC-7721 cells expressing shSNAI2 or shControl.

The above results of transformed hepatic tumor cells reflect that cells expressing shSNAI2 displayed higher anchorage-independent growth ability in HCC cells. To elucidate whether the phenotype is CSC-related, q-PCR was applied to test the CSC genes expression in these HCC cells. As shown in Fig 3A, among CSC-associated genes we tested, several genes, like EpCAM (*P<0*.*01*), ALDH1A1 (*P<0*.*0001*), CD24 (*P<0*.*0001*), CD56 (*P<0*.*01*) and CD133 (*P<0*.*05*), were significantly up-regulated in MHCCLM3-shSNAI2 other than MHCCLM3-shControl cells. Higher level of NANOG (*P = 0*.*0023*), ALDH1A1 (*P = 0*.*0035*), CD24 (*P = 0*.*0094*) and CD56 (*P = 0*.*0109*) were also observed in SMMC-7721 cells expressing shSNAI2 than shControl (S3A Fig). Consistent with shRNA results, over-expression of SNAI2 reduced mRNA levels of NANOG (*P = 0*.*0123*), ALDH1A1 (*P = 0*.*0075*), CD24 (*P = 0*.*0103*) and CD56 (*P = 0*.*01*) in SMMC-7721 cells (S3B Fig). HCC cells that have higher ability to effuse Hoechst 33342 were thought to have CSC characteristics [37]. So, FACS was applied to separate Hoechst 33342 high and low population of shSNAI2- and shControl-expressing MHCCLM3 cells [38]. Cells with lower efflux pump activity resulted in Hoechst 33342 high population (Fig 3B, encircled by red line), and cells with higher efflux pump activity resulted in Hoechst 33342 low population (Fig 3B, black line enclosed). Western blot was used to detect SNAI2 expression in cells with different efflux pump activity. More intriguingly, cells with brighter Hoechst 33342 staining expressed more SNAI2 than cells with lower level of Hoechst 33342, no matter in shControl or shSNAI2 expressing cells (Fig 3C, comparing line 1 vs. line 2, line 3 vs. line 4, line 5 vs. line 6). Moreover, SNAI2 inhibition could enhance efflux of Hoechst 33342: the percentage of Hoechst 33342 low cells increased (Fig 3D, left) and the percentage of Hoechst 33342 high cells decreased (Fig 3D, right) in shSNAI2 cells compared with the shControl cells. It suggested that SNAI2 inhibition could strengthen efflux ability of MHCCLM3 cells. It was reported that ABC transporters are the major efflux pumps on the surface of cancer cells [38], so q-PCR was used to detect the expression of ABC transporter genes in MHCCLM3 cells. Among key members of ABC transporters, expression of ABCB1 and ABCG2 were significantly up-regulated in shSNAI2-expressing MHCCLM3 cells (Fig 3E). Consistent with results in MHCCLM3 cells, up-regulation of ABCB1 and ABCG2 induced by SNAI2 inhibition could also been observed in SMMC-7721 (S3C Fig). It was deserved to be elucidated whether SNAI2, a transcriptional factor, directly regulated these genes through its transcriptional activity. The promoters of these genes were analyzed by the open-access JASPAR database [39], and potential binding sequences of SNAI2 were found in almost all of the above CSC and ABC genes. To confirm the prediction, DNA fragment of *ABCB1* gene promoter, which includes the 1 kb region upstream of transcriptional start site and exon 1 that carrying several potential SNAI2 binding sites, was cloned into a luciferase report pGL3-basic vector (Fig 3F). When co-transfected with *Renilla* into SMMC-7721 cells expressing shSNAI2 or shControl, the luciferse activity driven by the promoter of *ABCB1* gene could be dramatically induced accompanied by silence of SNAI2 (Fig 3G). Furthermore, western blot verified that protein level of ABCB1 increased when SNAI2 was inhibited (Fig 3H). In summary, these results suggested that endogenous SNAI2 suppressed CSC-related and ABC transporter genes expression and inhibited efflux pump activity of HCC cells.

### SNAI2 inhibition induces drug resistance of HCCs

**Fig 4:**
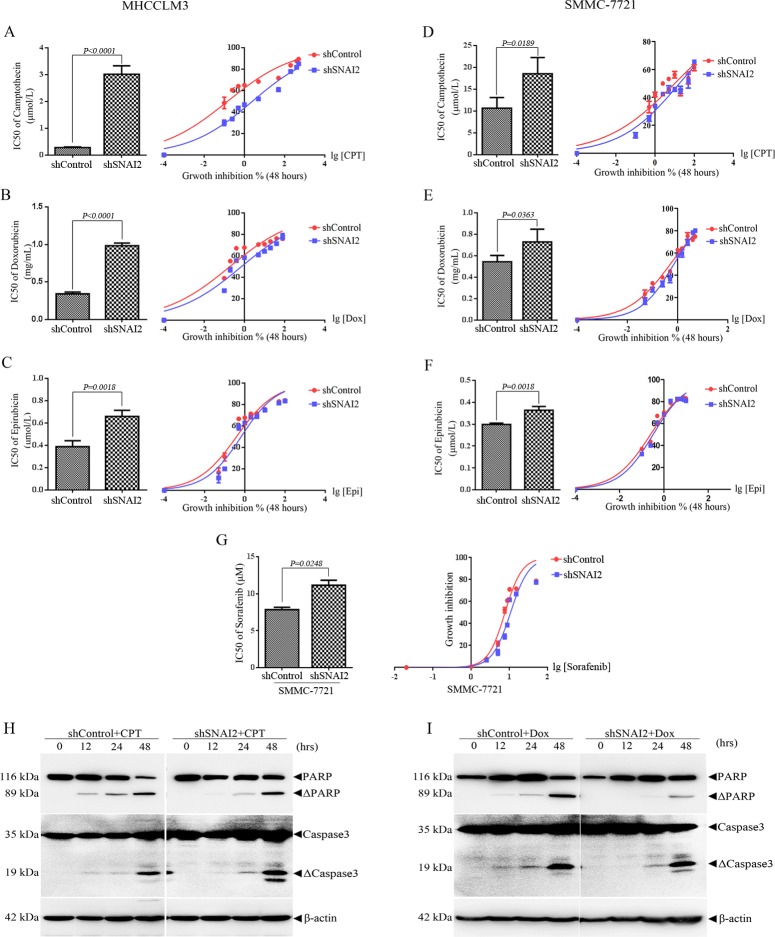
Inhibition of SNAI2 expression decreases the sensitivity to chemotherapy drugs of HCC cells. MHCCLM3 or SMMC-7721 cells were infected with lentivirus expressing shControl or shSNAI2 and selected by puromycin. (A-C) MHCCLM3 cells were treated with different concentrations of Camptothecin (CPT, A) Doxorubicin (Dox, B) and Epirubicin (Epi, C) for 48 hours, cell proliferation was measured by CCK-8 assay (right) and IC50 values (left) were calculated by GraphPad Prism 6.0 software. (D-G) SMMC-7721 cells were incubated with different concentrations of CPT (D), Dox (E), Epi (F), and Sorafenib (G) for 48 hours, cell growth (right) and IC50 values (left) were assessed as in MHCCLM3 cells. All values were represented as mean with bar as SD of three independent experiments and the *P* values were shown between two linked groups. (H/I) SMMC-7721 cells expressing shControl or shSNAI2 were treated with 10 μM CPT (H) or 0.5 mg/mL Dox (I) for different hours as shown, and Western blot was used to detect indicated proteins.

Cancer cells with higher efflux pump activity and ABC transporters expression always display enhanced resistance to chemotherapy drug [40]. So, MHCCLM3 and SMMC-7721 cells were treated with different concentrations of Camptothecin (CPT), Doxorubicin (Dox), and Epirubicin (Epi) for 48h, followed by cell counting kit 8 (CCK-8) assays. The results in Fig 4 revealed that cells with higher SNAI2 expression were more sensitive to chemotherapy drug than cells with lower SNAI2 expression. Inhibition of SNAI2 dramatically reduced the sensitivity of MHCCLM3 cells to CPT, Dox, and Epi (Fig 4A–4C). IC50 to CPT in shSNAI2-expressing MHCCLM3 cells increased 14 folds (Fig 4A). IC50 to Dox and Epi in shSNAI2-expressing MHCCLM3 cells increased 3.7 and 2 folds respectively, compared with shControl ones (Fig 4B and 4C). Similar results were also shown in SMMC-7721 cells. As shown in Fig 4D and 4E, SMMC-7721 cells were much more resistant to CPT (IC50 is 10.11 μM) and Dox (IC50 is 0.519 mg/mL), and inhibition of SNAI2 further reduced the sensitivity to CPT (IC50 up to 17.51 μM) and Dox (IC50 up to 0.7151 mg/mL). IC50 to Epi in SMMC-7721-shSNAI2 cells was 1.3 fold as in SMMC-7721-shControl cells (Fig 4F). Additionally, shSNAI2 expressing SMMC-7721 cells were more resistant to sorafenib, the most commonly used chemotherapy drug for HCC patients, than SMMC-7721-shControl cells (Fig 4G). Whether SNAI2 inhibition-induced sorafenib resistance related to the alteration of ABC transporters as ABCG2 remains to be elucidated [41]. Subsequently, SMMC-7721 cells expressing shControl or shSNAI2 were subcutaneously inoculated into BALB/c nude mice, and the effect of SNAI2 expression on drug sensitivity of CPT was analyzed *in vivo*. Two weeks after inoculation, 20 mg/kg CPT was injected intraperitoneally as described in Materials and Methods. Admission of CPT did not show toxic since the body weight of mice did not change obviously during the treatment. When tumor burden in saline group reached the maximum limitation, the experiment had to be terminated due to ethical consideration. Mice were euthanized and tumors were dissected and weighted. The tumor mass of shControl or shSNAI2 xenografts in saline treatment group showed no difference, which was consistent with the fact that SNAI2 expression did not influence HCC cell proliferation (S4A and S4B Fig). No matter in shControl or shSNAI2 xenografts, tumor volume and weight were dramatically less in CPT treatment group than those in saline group, which indicated CPT treatment significantly inhibited tumor growth (S4A and S4B Fig). Notably, tumors of shSNAI2 xenograft in CPT treatment group weighted much heavier than those shControl xenograft at the end of therapy (S4B Fig, two groups in right). It suggested SNAI2 inhibition endued drug resistance after chemotherapy (S4A and S4B Fig). In line with this notion, when SMMC-7721 cells were treated with 10 μM CPT for different hours, Caspase-3 activation with the cleavage of its substrate poly-(adenosine diphosphate ribose) polymerase (PARP) were induced 12 hours earlier in SMMC-7721-shControl cells than in SMMC-7721-shSNAI2 cells (Fig 4H). Moreover, the activation of Caspase-3 and the cleavage of PARP could be detected at 12 hours after DOX treatment in shControl cells, while same phenotype delayed to the 24th hour in shSNAI2 SMMC-7721 cells (Fig 4I). Of note, the cleavage of PARP at the 48th hour was much more significant in shControl than shSNAI2 SMMC-7721 cells (Fig 4I). Collectively, these results suggested that SNAI2 contributed to chemotherapy-induced apoptosis and drug sensitivity of HCC cells.

### Verapamil, inhibitor of ABC transporter, recovers the sensitivity of MHCCLM3 to chemotherapy drugs

**Fig 5:**
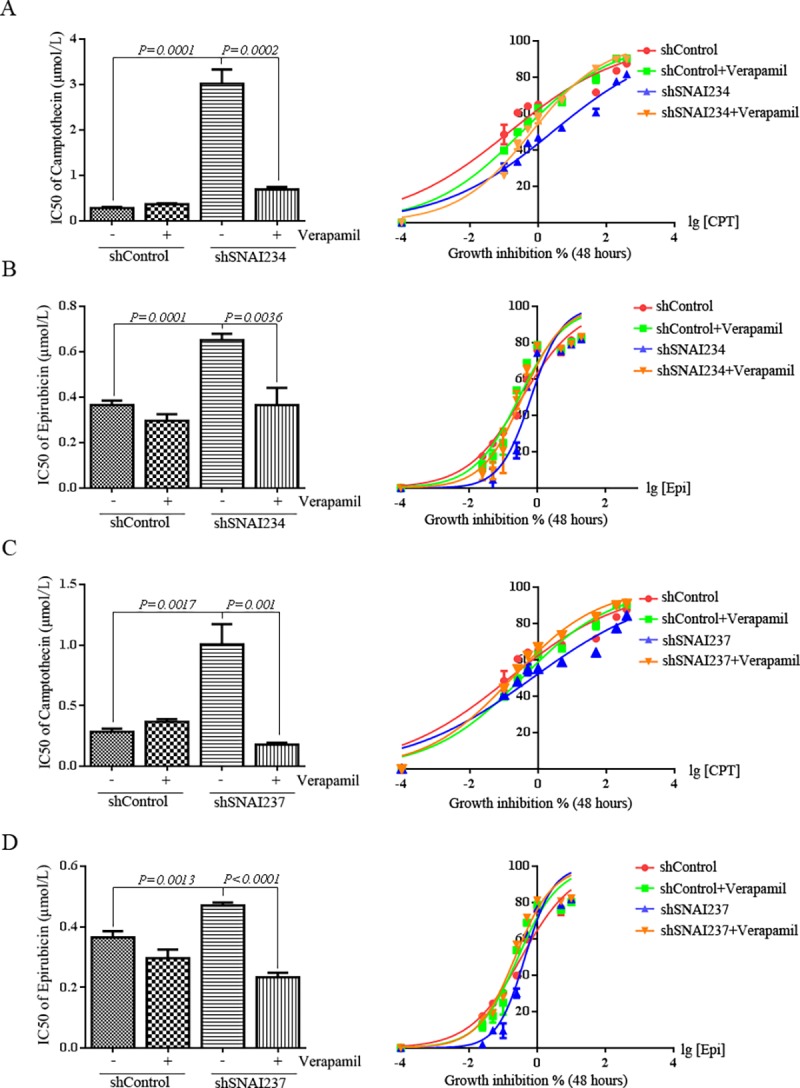
Verapamil, inhibitor of ABC transporter, recovers the sensitivity of MHCCLM3 to chemotherapy drugs. MHCCLM3 cells expressing shSNAI234 (A-B) or shSNAI237 (C-D) were pretreated with 1 μM Verapamil, then CPT (A/C) or Epi (B/D) was added at different concentrations for 48 hours, cell growth was tested by CCK-8 assay (right), and IC50 values (left) of CPT/Epi were calculated by GraphPad Prism 6.0 software. All experiments were repeated three times with similar results. All values were represented as mean with bar as SD of three independent experiments, and the *P* values were measured between two linked groups.

To further elucidate the role of ABC transporters in SNAI2 inhibition induced multidrug resistance, verapamil was used to abolish efflux activity of ABC transporters. SNAI2 inhibition could dramatically increase IC50 to CPT in MHCCLM3 cells (Fig 5A). Comparing with shSNAI234-expressing MHCCLM3 cells, IC50 to CPT reduced 4 folds (Fig 5A) and IC50 to Epi decreased 1.8 folds (Fig 5B) in shSNAI234-expressing cells pre-treated with verapamil. To exclude the off-target effect of single shSNAI2, another shRNA of SNAI2, shSNAI237 was transfected into MHCCLM3 cells. IC50 to CPT increased 1.9 folds and IC50 to Epi increased 1.8 folds in MHCCLM3-shSNAI237 cells compared with MHCCLM3-shControl cells. Verapamil incubation almost completely inhibited shSNAI237-induced IC50 alteration to CPT or Epi (Fig 5C and 5D). In total, these results manifested that inhibition of ABC transporters could efficiently reverse shSNAI2-induced multidrug resistance in MHCCLM3 cells.

## Discussion

Although SNAI2 is regarded as a cancer promoter factor based on the facts that SNAI2 promotes survival, suppresses apoptosis and drives EMT transition and metastasis in many types of cancer [7, 18, 19]. Herein, our data show that SNAI2 behaves as a tumor suppressor by increasing drug sensitivity via inhibiting ABC transporter genes in HCC cells based on the following evidences: First, inhibition of SNAI2 expression induces proliferation of non-cancer hepatic cells, promotes anchorage-independent growth of HCC cells and up-regulates CSC genes as well. Second, inhibition of SNAI2 expression initiates drug resistance *in vitro* and *in vivo* even though it does not influence metastatic ability of HCC cells. Third, reducing SNAI2 level enhances efflux of Hoechst 33342 and up-regulates ABC transporter genes, and pretreatment of ABC inhibitor varapamil can attenuate the multi-drug resistance triggered by SNAI2 inhibition.

Although various treatments have greatly improved the survival rate of HCC patients, relapse as a result of metastasis and resistance to chemotherapy drugs remains unsolved. There are accumulating evidences that CSC may be the main reason of aggressive behavior and drug resistance in HCC cells [42]. The roles of SNAI2 in CSC remain controversial. In normal mammary epithelial cells, SNAI2 and Sox9 have been reported to work together to transform the normally differentiated breast cells into progenitor/stem cells [18]. However, Ye X. et al have reported normal mammary stem cells expressing high level of SNAI2 are deficient in tumor-initiating ability in mice [27]. Our data showed that SNAI2 knockdown could reinforce anchorage-independent growth of HCC cells in tumor sphere culture and soft agar clone formation assay. CSCs can be identified by specific antigens expressed on the cell surface. EpCAM, CD133, CD56, ALDH1A1, CD24, CD44 and so on are regarded as CSC markers of HCC cells [42, 43]. Our subsequent q-PCR showed that multiple CSC-related markers, such as ALDH1A1, CD24, and CD56, were up-regulated in HCC cells. In mammary epithelial cells, SNAI2 is enriched in EpCAM-expressing cells while in some of the pulmonary metastatic high-grade carcinomas, the expression of epithelial marker EpCAM is downregulated and so does the SNAI2 expression [27]. Of note, EpCAM, which is a mammary epithelial marker, is regarded as a stemness-related marker in HCC cells [27, 42]. Our results indicated that SNAI2 knockdown promoted the expression of CSC marker EpCAM in HCC cells, which was consistent with the enhanced anchorage-independent growth. Cells with higher ability to efflux of Hoechst 33342 were thought to have CSC characteristics in HCC cells [37]. Our data demonstrated that SNAI2 inhibition could enhance the efflux pump ability of Hoechst 33342 and induce expression of multiple ABC transporter genes. Efflux pump ability and ABC transporter gene expression closely related to drug resistance of cancer cells [38, 44], which is the main challenge of cancer therapy. Previous data suggest that the SNAI2 expression positively relates with drug resistance. Chang T.H. et al have reported that SNAI2 contributes to the resistance to gefitinib in NSCLC and maybe a potential therapeutic target for treating EGFR TKIs-resistant NSCLC [22]. In DU145 prostate cancer cells, over-expression of SNAI2 increases drug resistance to doxorubicin and SNAI2 down-regulation sensitized DU145 cell line to chemotherapeutic drugs by increasing PTEN expression [45]. Knockdown of SNAI2 expression in ovarian cells that are resistant to cisplatin can reduce the migratory and invasive capacities and increase cellular sensitivity to cisplatin relative to controls [46]. In CML, Bcr-Abl can transcriptionally drive the expression of SNAI2 and post-transcriptionally stabilize SNAI2 protein, and SNAI2 mediates Bcr-Abl-T315I-driven resistance of leukemic progenitor to imatinib mesylate through the repression of pro-apoptotic Puma [21]. Intriguingly, our data indicated SNAI2 was negatively related with drug resistance, and inhibition of SNAI2 induced multidrug resistance and resisted chemotherapy induced-apoptosis of HCCs *in vitro*. Although CPT could effectively inhibit growth of SMMC-7721 xenograft, several cycles of CPT treatment endowed drug resistance of shSNAI2 xenograft and shSNAI2 but not shControl tumors overcame the CPT-mediated growth inhibition *in vivo*. Consistent with *in vivo* data, HCC patients from TCGA database with low SNAI2 expression were more likely to have advanced stage tumors (S4C Fig). It remains to be elucidated whether shSNAI2 inhibition-induced drug resistance is correlated with tumor malignancy. ABC transporters are reported as the major reason for multidrug resistance of cancer [47]. ABC transporter can transport a wide range of chemotherapeutic drugs including the anthracyclines, vinca alkaloids, taxanes, and epipodophyllotoxins [47, 48]. In present study, we firstly certified that SNAI2 inhibition up-regulated expression of ABC transporter proteins, as ABCB1 and ABCG2. Through JASPAR database, the promoters of these genes were analyzed and several potential binding sites of SNAI2 were identified. Luciferase assay further confirmed the transcriptional repression of SNAI2 on ABC transporter genes like ABCB1. Moreover, pretreatment of verapamil, the inhibitor of ABC proteins, effectively rescued the sensitivity of HCC cells to chemotherapy drugs. Our data demonstrated for the first time that SNAI2 played negative roles in multi-drug resistance through inhibition of ABC transporter genes in HCC cells, even through these discoveries remain to be further investigated in human samples.

## Conclusions

In conclusion, our study suggests the new biological function of SNAI2 in HCC cells and certifies it’s tightly junction with multidrug resistance rather than migratory capability. Our results provide new clues to study the molecular mechanisms of development of HCC and would shed new insights on treatment of liver cancer.

## Supporting Information

S1 Fig Effects of SNAI2 expression on proliferation of non-cancer HL-7702 cells and E-cadherin expression of MHCCLM3 cells. (A-B) Human immortal hepatic HL-7702 cells were infected with shSNAI2/shControl lentivirus. (A) Western blot were used to measure expression of SNAI2 with β-actin as internal control. (B) Cell proliferation was assessed by CCK-8 assay and relative growth rate calculated by GraphPad Prism 6.0 software (C) Western blot were used to test expression of indicated proteins in MHCCLM3 cells transient transfected with shRNAs or plasmids as shown.
(TIF)

S2 Fig Ectopic SNAI2 expression has no effect on metastatic properties of SMMC-7721 cells. SMMC-7721 cells stably expressing SNAI2 or control vector were used. (A) SNAI2 expression was measured by Q-PCR (up) and Western blot (down). (B-C) Tumor sphere culture was performed as described in Materials and Methods. Representative images of tumor spheres were shown (B), and the number of spheres (>100 μm in diameter) were counted and calculated by GraphPad Prism 6.0 software (C). (D-H) Metastatic abilities were detected in SMMC-7721 cells with ectopic SNAI2 expression. Scratch wound healing ability was tested (D) and relative migration distance was measured and calculated by GraphPad Prism 6.0 software (E). Migration ability was investigated by xCELLigence RTCA assays (F) or in vitro transwell migration assay (G/H) respectively. Representative images of migration cells (G) were shown, and migration cell numbers were counted and calculated by GraphPad Prism 6.0 software (H). All values were represented as mean with bar as SD of three independent experiments, and student *t*-test was used to compare linked groups as shown.
(TIF)

S3 Fig Alteration of SNAI2 expression influences CSC and ABC transporter gene expression of SMMC-7721 cells. SMMC-7721 cells stably expressing shSNAI2/shControl (A/C) or vector/SNAI2 plasmids (B) were used. Q-PCR was applied to detect mRNA levels of indicated genes in SMMC-7721 cells. Relative expression of indicated genes was normalized with *GAPDH* mRNA. All values were represented as mean with bar as SD of three independent experiments and the *P* values were shown between two linked groups.
(TIF)

S4 Fig Inhibition of SNAI2 induces CPT resistance of SMMC-7721 xenograft *in vivo*. (A-B) SMMC-7721-shControl/–shSNAI2 cells were transplanted in contralateral flanks of nude mice for 2 weeks. Then mice were intraperitoneally injected with 20 mg/kg Camptothecin (CPT) for 3 continuous courses, and each course included continuous three days’ injection and one day’s break. (A) The representative tumor masses were photographed at the end of CPT treatment. (B) The tumor weights were measured and calculated by GraphPad Prism 6.0 software. The values were represented as mean with bar as SD of four mice each group and the *P* values were shown between linked groups. (C) Percentage plot of patients from TCGA database that segregated by low (upper quartile) or high expression of SNAI2 with AJCC stage I-IV hepatocelluar carcinoma.
(TIF)
