# Supplementary figures and images for: Inhibition of Snail Family Transcriptional Repressor 2 (SNAI2) Enhances Multidrug Resistance of Hepatocellular Carcinoma Cells
Source: PLoS One. 2016 Oct 19;11(10):e0164752. doi: 10.1371/journal.pone.0164752 (PMC5070735; doi:10.1371/journal.pone.0164752)

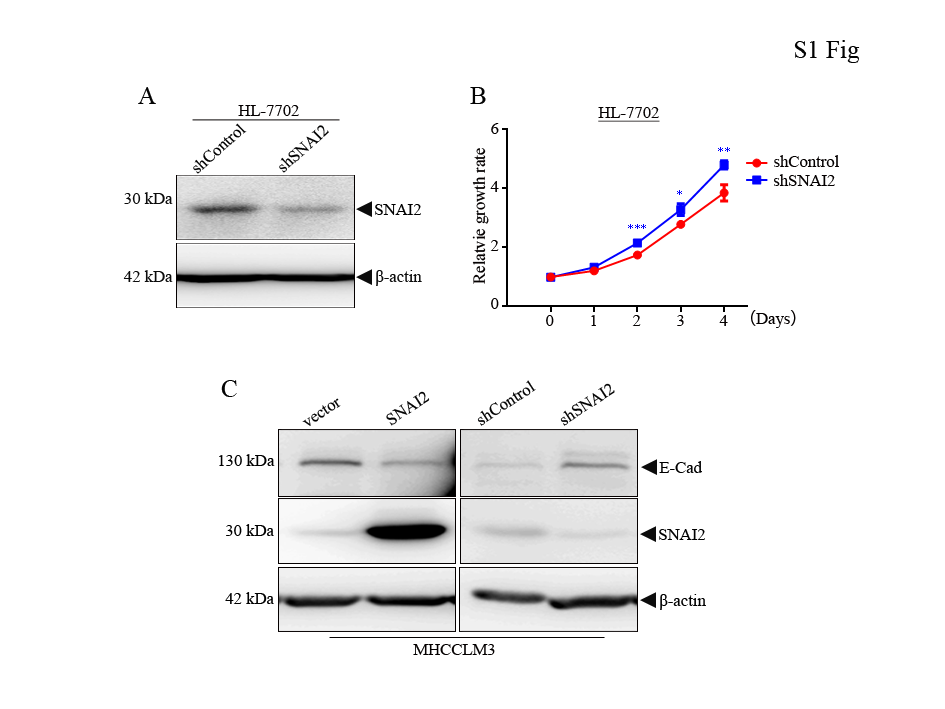

Supplement: S1 Fig — (A-B) Human immortal hepatic HL-7702 cells were infected with shSNAI2/shControl lentivirus. (A) Western blot were used to measure expression of SNAI2 with β-actin as internal control. (B) Cell proliferation was assessed by CCK-8 assay and relative growth rate calculated by GraphPad Prism 6.0 software (C) Western blot were used to test expression of indicated proteins in MHCCLM3 cells transient transfected with shRNAs or plasmids as shown. (TIF) [file pone.0164752.s001.tif]

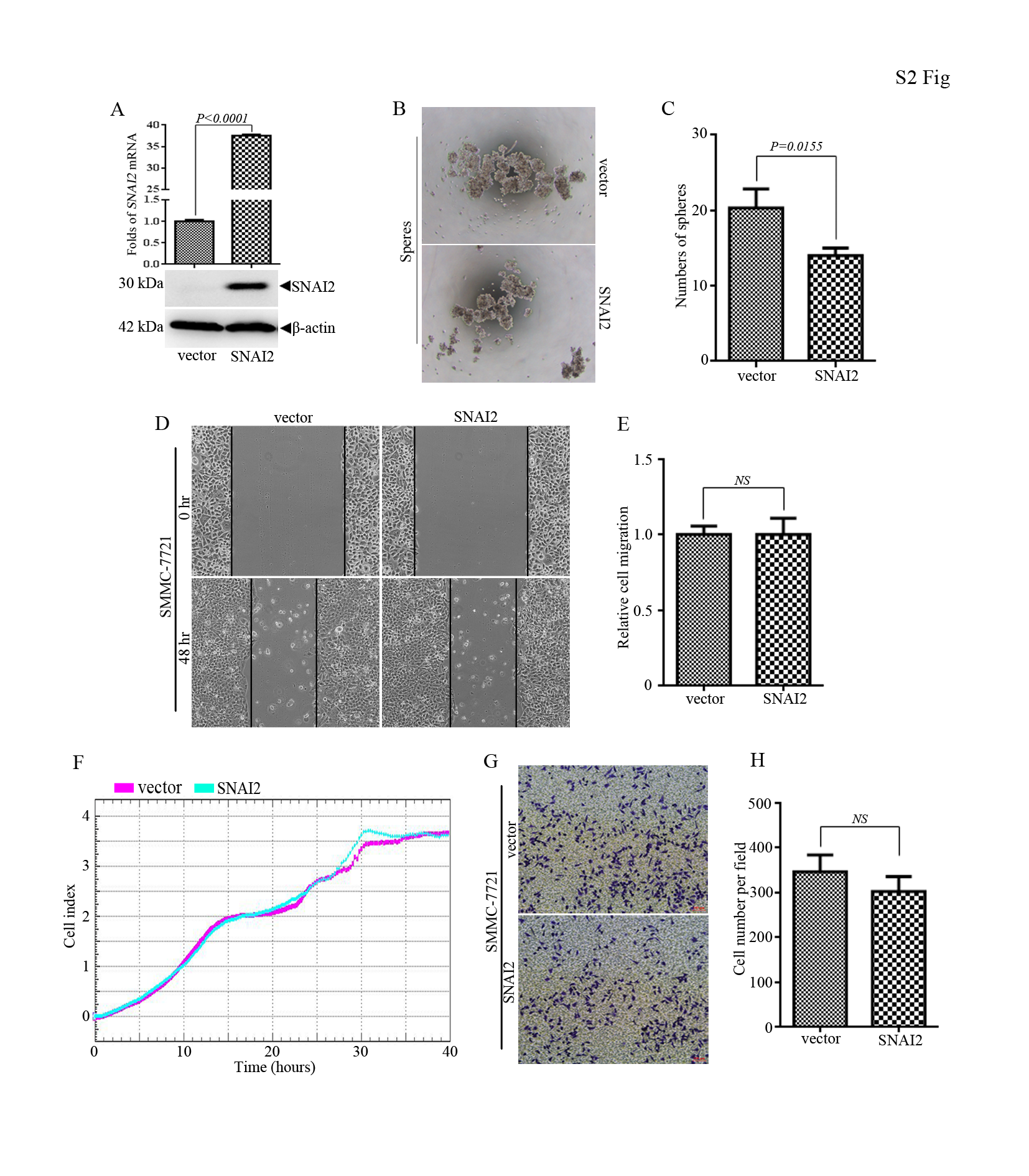

Supplement: S2 Fig — SMMC-7721 cells stably expressing SNAI2 or control vector were used. (A) SNAI2 expression was measured by Q-PCR (up) and Western blot (down). (B-C) Tumor sphere culture was performed as described in Materials and Methods. Representative images of tumor spheres were shown (B), and the number of spheres (>100 μm in diameter) were counted and calculated by GraphPad Prism 6.0 software (C). (D-H) Metastatic abilities were detected in SMMC-7721 cells with ectopic SNAI2 expression. Scratch wound healing ability was tested (D) and relative migration distance was measured and calculated by GraphPad Prism 6.0 software (E). Migration ability was investigated by xCELLigence RTCA assays (F) or in vitro transwell migration assay (G/H) respectively. Representative images of migration cells (G) were shown, and migration cell numbers were counted and calculated by GraphPad Prism 6.0 software (H). All values were represented as mean with bar as SD of three independent experiments, and student t-test was used to compare linked groups as shown. (TIF) [file pone.0164752.s002.tif]

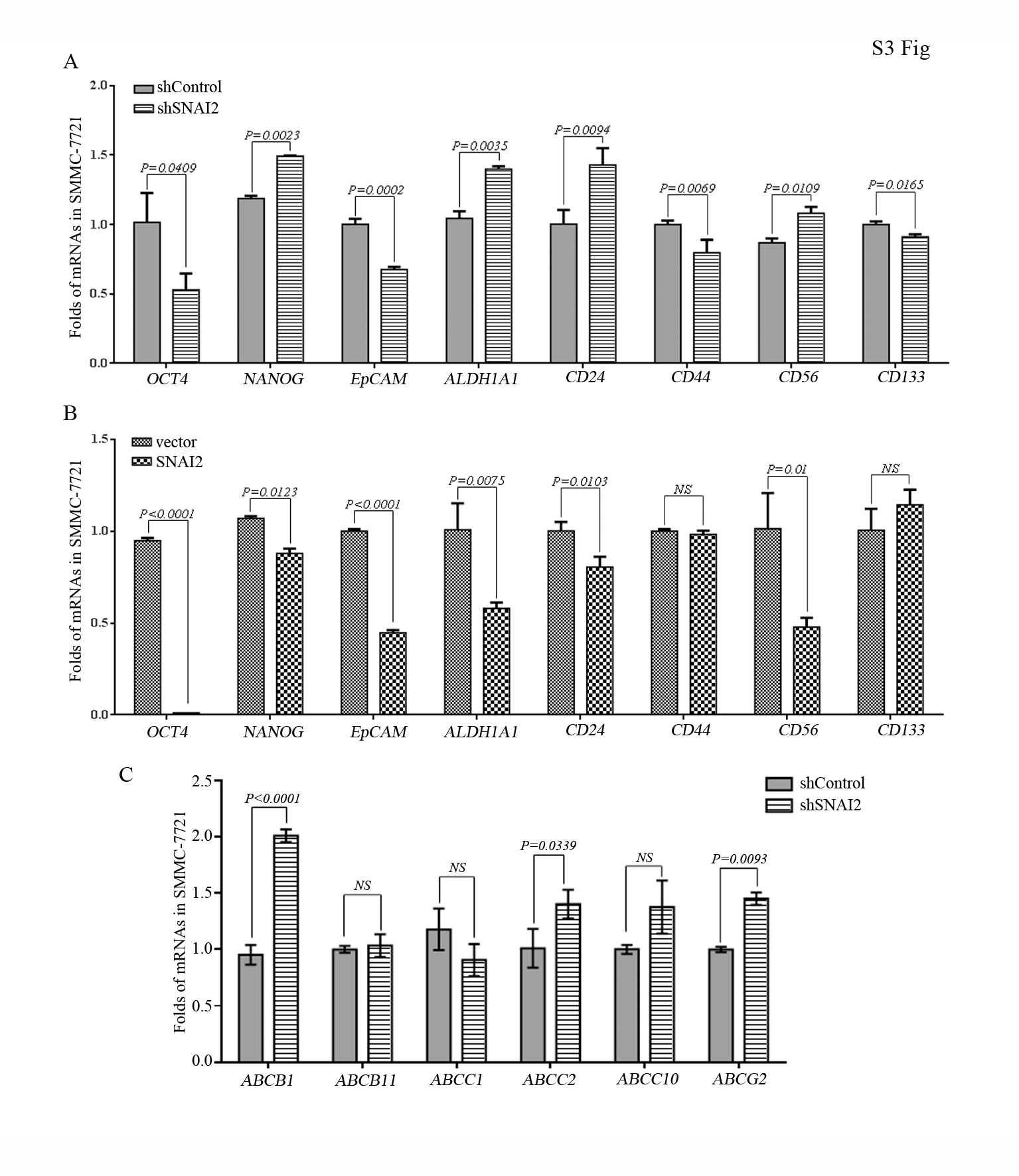

Supplement: S3 Fig — SMMC-7721 cells stably expressing shSNAI2/shControl (A/C) or vector/SNAI2 plasmids (B) were used. Q-PCR was applied to detect mRNA levels of indicated genes in SMMC-7721 cells. Relative expression of indicated genes was normalized with GAPDH mRNA. All values were represented as mean with bar as SD of three independent experiments and the P values were shown between two linked groups. (TIF) [file pone.0164752.s003.tif]

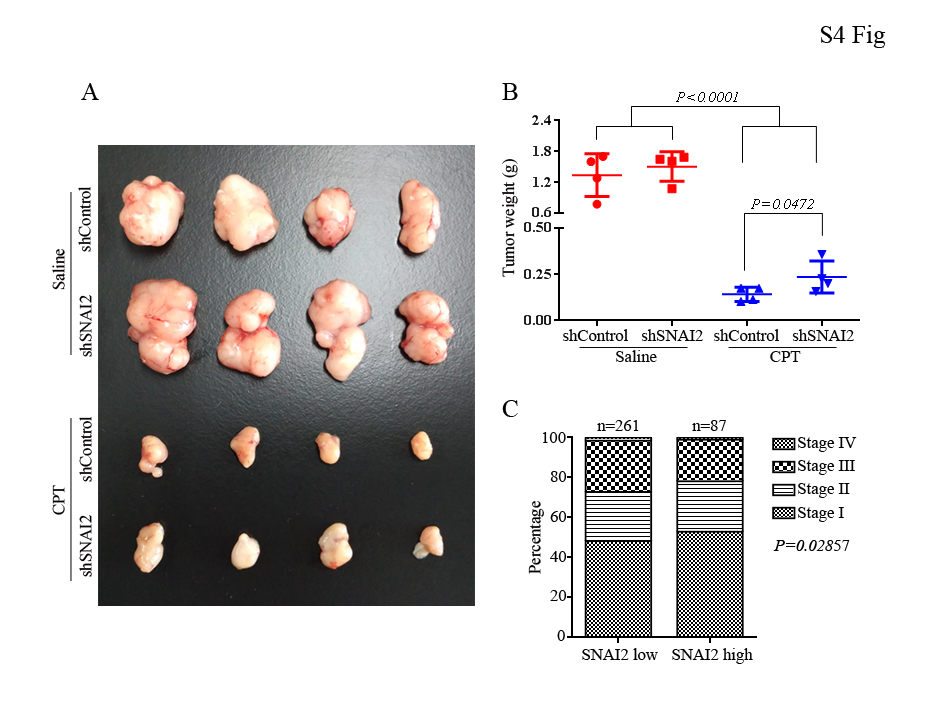

Supplement: S4 Fig — (A-B) SMMC-7721-shControl/–shSNAI2 cells were transplanted in contralateral flanks of nude mice for 2 weeks. Then mice were intraperitoneally injected with 20 mg/kg Camptothecin (CPT) for 3 continuous courses, and each course included continuous three days’ injection and one day’s break. (A) The representative tumor masses were photographed at the end of CPT treatment. (B) The tumor weights were measured and calculated by GraphPad Prism 6.0 software. The values were represented as mean with bar as SD of four mice each group and the P values were shown between linked groups. (C) Percentage plot of patients from TCGA database that segregated by low (upper quartile) or high expression of SNAI2 with AJCC stage I-IV hepatocelluar carcinoma. (TIF) [file pone.0164752.s004.tif]
